# Computed tomography-guided percutaneous biopsy of subcentimeter lung noduless

**DOI:** 10.1590/0100-3984.2024.0046-en

**Published:** 2024-11-18

**Authors:** Penélope Sánchez Teixeira, Almir Galvão Vieira Bitencourt, Jefferson Luiz Gross, Rubens Chojniak, Soraia Quaranta Damião, Paula Nicole Vieira Pinto Barbosa

**Affiliations:** 1 Radiology Department, A.C.Camargo Cancer Center, São Paulo, SP, Brazil

**Keywords:** Image-guided biopsy/adverse effects, Tomography, X-ray computed, Multiple pulmonary nodules, Lung neoplasms., Biópsia guiada por imagem/efeitos adversos, Tomografia computadorizada, Nódulos pulmonares múltiplos, Neoplasias pulmonares.

## Abstract

**Objective:**

To assess the diagnostic success rate and complications of computed tomography (CT)-guided percutaneous biopsy in pulmonary nodules < 10 mm in diameter.

**Materials and Methods:**

This was a retrospective, single-center study involving the review of medical records, images, and chest CT reports related to 115 patients who underwent percutaneous CT-guided biopsy of < 10 mm pulmonary nodules between July 2015 and January 2019.

**Results:**

Nodule diameter on the longest axis ranged from 4 mm to 9 mm, with a mean size of 7.7 mm. The mean age of the patients at the time of the procedure was 61 years, and 54.7% were women. Of the 115 nodules evaluated, 77 (67.0%) were solid and 55 (47.8%) were located in the lower lobes. The mean distance traversed by the needle in the lung parenchyma was 20 mm (range, 0-70 mm), and, in most cases, the biopsy was not performed with the patient in the biopsy-side-down lateral position. The diagnostic success rate was 93.0%. The most common complications were alveolar hemorrhage (in 36.5% of cases) and pneumothorax (in 24.3%).

**Conclusion:**

The data suggest that CT-guided percutaneous biopsy of < 10 mm pulmonary nodules has a high diagnostic success rate and an acceptable rate of complications.

## Abstract (pt)

**Objetivo:**

Avaliar a taxa de sucesso diagnóstico e complicações da biópsia percutânea por fragmentos guiada por tomografia computadorizada (TC) em nódulos pulmonares menores que 10 mm.

**Materiais e Métodos:**

Estudo retrospectivo, unicêntrico, com base em revisão de prontuários, imagens e laudos de tomografia de tórax em 115 pacientes submetidos a biópsia percutânea por fragmentos guiada por TC em nódulos pulmonares menores que 10 mm, entre julho de 2015 e janeiro de 2019.

**Resultados:**

Os nódulos variaram de 4 a 9 mm, com tamanho médio de 7,7 mm no maior eixo do nódulo. A idade média dos pacientes no procedimento foi 61 anos, sendo 54,7% do sexo feminino. Os nódulos sólidos foram os mais prevalentes (67,0%), a maioria estava localizada nos lobos inferiores (47,8%), a distância média percorrida no parênquima pulmonar foi 20 mm (variando de 0 a 70 mm) e na maior parte dos casos a biópsia não foi realizada em decúbito restritivo. A taxa de sucesso diagnóstico foi 93,0%. As complicações mais frequentes foram hemorragia alveolar (36,5%) e pneumotórax (24,3%).

**Conclusão:**

Os dados sugerem que a biópsia pulmonar percutânea guiada por TC de nódulos pulmonares menores de 10 mm apresentou alta taxa de sucesso diagnóstico e incidência de complicações aceitável.

## INTRODUCTION

With the significant increase in the use of computed tomography (CT), there has been constant growth in the application of imaging protocols aimed at screening for lung cancer in the early stages, using reduced doses of radiation. These protocols have enabled the detection of an increasing number of small pulmonary nodules, which represents a substantial challenge for radiologists, who must attempt to differentiate between malignant and benign lesions in the quest for early diagnosis and guidance on the selection of specific treatments^**(1)**^. In addition to its screening function, CT has become the imaging modality of choice to guide transthoracic lung biopsy^**(2)**^.

For the study of small pulmonary lesions, percutaneous transthoracic lung biopsy guided by CT with a core needle has shown to be an effective, reproducible, and acceptable procedure in relation to conventional methods such as sputum cytology, thoracotomy, thoracoscopy, and bronchoscopy^**(3-5)**^. It provides better guidance and precision for the procedure, assists in differentiating between a solid area and areas that are less solid and more heterogeneous, such as areas of necrosis/liquefaction, and thus facilitates the collection of material that is more representative and more suitable for histological analysis. Although open surgical biopsy is still considered the standard in some places, that is an invasive procedure that can be result in significant morbidity or even death^**(6)**^. In addition, CT-guided lung biopsy facilitates the diagnosis of a variety of conditions because it is particularly useful in clarifying the nature of pulmonary nodules and masses, distinguishing scar tissue from tumor recurrence, and histologically characterizing advanced diseases. It is also valuable for determining the molecular profile of lesions, in order to refine the treatment with chemotherapy or targeted therapies^**(6)**^.

Biopsy with CT guidance is indicated even in cases where lesions could be surgically removed, offering a way to avoid unnecessary surgical procedures, especially for lesions that are likely benign or nonspecific. It has also increasingly become an option for immunocompromised patients with consolidations or abscesses when other diagnostic methods have failed^**(7-9)**^.

## MATERIALS AND METHODS

### Study design

This was a retrospective, single-center study, based on the review of medical records, images, and reports related to patients undergoing CT-guided percutaneous biopsy of pulmonary nodules < 10 mm, for diagnostic purposes, between July 2015 and January 2019 at a referral center for cancer in Brazil. The study was approved by the local research ethics committee, and all patients gave written informed consent.

### Population

We evaluated 115 CT-guided biopsies of pulmonary nodules < 10 mm (range, 4-9 mm). The biopsies were performed with an 18 G or 20 G needle and a 17 G or 19 G coaxial system, respectively. All of the patients had been referred for the diagnosis of primary, metastatic, or inflammatory lung lesions.

### Data collection

A standardized data collection form was employed, and electronic medical records were reviewed for patient sociodemographic data; clinical history; characteristics of the lesion to be biopsied, including maximum diameter on the longest axis, location (upper, middle, or lower lobe), and consistency (solid, semisolid, or ground glass); the final biopsy report; pathological findings; and clinical follow-up data. To ensure the integrity of the results, we excluded patients for whom the documentation was incomplete or for whom images were unavailable.

### Procedure

The biopsy procedures were performed by interventional radiology residents under the direct supervision of senior physicians who were specialists in the field. Images were acquired in a high-speed helical CT scanner (General Electric, Milwaukee, WI, USA), configured for low-dose images (120 kVp, 30 mAs) and a slice thickness of 3 mm. The precise location of the nodule was determined by using laser light from the gantry and a radiopaque marker in the area of interest.

The optimal patient position for lung biopsy was determined on a case-by-case basis, in accordance with strict positioning criteria. Options included placing the patient in the lateral position, either biopsy side down or biopsy side up.

The coaxial technique was applied in all cases, with a 17 G or 19 G coaxial needle (TruGuide; Becton, Dickinson and Company, Tempe, AZ, USA) and another 18 G or 20 G core needle (Tru-Cut; Becton, Dickinson and Company). The appropriate needle length was determined on the basis of the distance between the lesion and the skin, as measured on CT images reviewed during the planning of the procedure.

After needle insertion, images with a slice thickness of 3 mm were obtained to verify the position of the needle tip in the target lesion. After confirming the appropriate positioning of the needle tip in the lesion, a semi-automatic cutting needle (18 G or 20 G) was introduced, and three to four fragments of the lesion were removed. If the patient had any complications during the biopsy, a situation that was not observed in our sample, that number of fragments would not have been removed. After the needle had been removed (i.e., immediately after the procedure), a low-dose CT examination (120 kVp, 10 mAs, 7 mm slice thickness) was performed to identify possible complications such as alveolar hemorrhage, hemothorax, and pneumothorax. The patients were subsequently monitored and underwent follow-up CT at two specific time points: in the first hour after the end of the procedure and again three hours after.

### Statistical analysis

Exploratory data analysis was carried out using summary measures (mean, standard deviation, minimum, first quartile, median, third quartile, maximum, frequency, and percentage). Comparisons between groups were made with the Mann-Whitney test for numeric variables and with the chi-square or Fisher’s exact test for categorical variables. The Shapiro-Wilk test was used in order to test the normality of numerical variables (age, size, and distance), and none of them showed a normal distribution^**(10,11)**^. The significance level adopted was 5%. All analyses were performed with the R software (R Core Team 2019).

## RESULTS

We evaluated 115 patients who underwent lung biopsy of nodules < 10 mm using the coaxial system guided by CT. As shown in Table 1, the mean age was 61.1 years (range, 24-85 years), and 63 (54.8%) of the patients were women. The mean nodule diameter on the longest axis was 7.7 mm (range, 4-9 mm). The mean distance covered by the needle in the lung parenchyma until reaching the lesion was 20.5 mm, and that distance ranged from 0 mm (for subpleural lesions) to 70 mm.

**Table 1:** Distribution of the sample regarding age, nodule size, and distance covered from the parenchyma along the biopsy needle path.

Variable	Mean	Standard deviation	Minimum	Maximum
Age (years)	61.1	13.6	24.0	85.0
Size (mm)	7.7	1.4	4.0	9.0
Distance (mm)	20.5	15.0	0.0	70.0

Of the 115 nodules evaluated, 77 (67.1%) were solid, 10 (8.6%) were ground glass and 28 (24.3%) were semisolid. Fifty-five nodules (47.8%) were located in the lower lobes, 38 (33.0%) were located in the upper lobes, and 22 (19.1%) were located in the middle lobe or lingula. Biopsies were performed in the biopsy-side-down lateral position in 45 cases (39.1%) and in the biopsy-side-up lateral position in 70 (60.8%). All procedures were performed using 18 G or 20 G thick needles and 17 G or 19 G coaxial systems. No significant association was observed with the study results (*p* = 0.791), as detailed in Table 2.

**Table 2:** Distribution and percentage of nodule density, needle diameter, and patient position.

Characteristic	Category	(N = 115) n (%)
Density	Solid	77 (67.0)
	Semisolid	28 (24.3)
	Ground glass	10 (8.7)
Needle	18 G	86 (74.8)
	20 G	29 (25.2)
Lateral positioning	No	70 (69.9)
	Yes	45 (39.1)

Sixty-three nodules (54.7%) were identified as malignant, including one (1.6%) with a squamous pattern and atypia, 10 (15.9%) that were adenocarcinomas with a lipid pattern, 30 (47.6%) that were adenocarcinomas without a lipid pattern, one (1.6%) that was a lymphoma, and 21 (33.3%) that were metastases.

Nonspecific histological findings, characterized by lung parenchyma with septal fibrosis, inflammatory processes, and foci of anthracosis, were obtained in 21 (18.2%) of the cases. Of those 21 cases, seven (33.3%) were lost to follow-up and seven (33.3%) remained stable. Another four (19%) showed disease progression, evidenced by the enlargement of the nodule or the appearance of new suspicious nodules, and three (14.2%) underwent surgery, resulting in two diagnoses of metastasis and one of benign granuloma. Samples with satisfactory results, suitable for diagnosis, were obtained from the remaining 94 lesions (81.7%). Regarding the diagnosis of benign disease, observed in 31 (26.9%) cases, and specific histopathological confirmation of benignity was achieved in 18 (58.0%) of those cases. Those 18 cases comprised one case of hamartoma, one case of intraparenchymal lymph node, five cases of granuloma, nine cases of organizing pneumonia, and two cases of fungal infection. The remaining 13 cases (41.9%) were confirmed as benign on the basis of regression of the lesion under conservative therapy or on the basis of stability or disappearance of the nodule on subsequent examinations.

Of the lesions of the lung parenchyma with histopathological results, considered false-negative cases, two were surgically resected. The first, located in the anterior basal segment of the lower lobe, turned out to be a typical carcinoid tumor. The second, located in the apical segment of the upper lobe, was diagnosed as a metastasis from a clear cell renal cell carcinoma. A lesion that had a true-negative biopsy result, with histopathology indicating interstitial fibrosis and a chronic inflammatory process with macrophages and no atypia, was also surgically resected. The surgical result confirmed the presence of granuloma with caseous necrosis.

A repeat biopsy was not performed in any of the cases in this study. However, in situations of discordance among the clinical, radiological, or histological findings, it would be considered a viable option.

The overall sensitivity, specificity, positive predictive value, and negative predictive value for the diagnosis of malignancy were 90.6%, 97.5%, 98.5%, and 84.7%, respectively. The diagnostic success rate was 93.0%.

**Figure 1:**
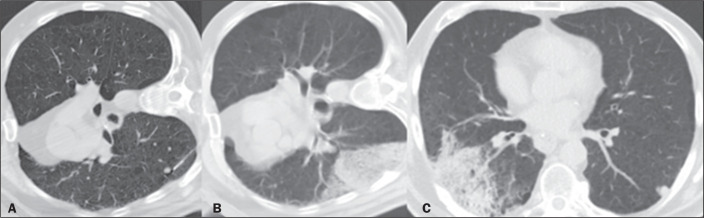
A: Pulmonary nodule with regular margins, measuring 4 mm, with needle insertion into the lung parenchyma. B: Alveolar hemorrhage after collection on immediate follow-up CT. C: Follow-up CT at three hours after the procedure, showing stabilization of the hemorrhage.

**Figure 2:**
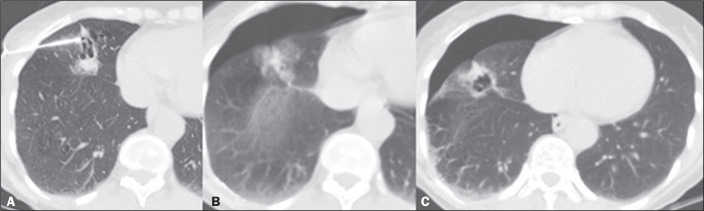
A: Cavitary pulmonary nodule with irregular margins, measuring 8 mm, with needle insertion into the lung parenchyma. B: Small pneumothorax after collection on immediate follow-up CT. C: Follow-up CT at three hours after the procedure, showing stabilization of the pneumothorax.

**Figure 3:**
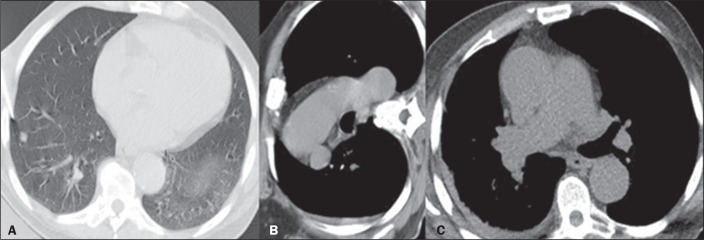
A: Pulmonary nodule with regular margins, measuring 5 mm, with needle insertion into the lung parenchyma. B: Small hemothorax after collection on immediate follow-up CT. C: Follow-up CT at three hours after the procedure, showing stabilization of the hemothorax.

Of the 115 patients evaluated, 53 (46.1%) did not experience any adverse events, 49 had one complication, and 13 (11.3%) had two complications. The most common complication was alveolar hemorrhage, which occurred in 42 cases (36.5%), of which 36 (85.7%) were considered mild (Figure 1). The second most common complication was pneumothorax, seen in 28 cases (24.3%), of which 23 (82.1%) were classified as mild (Figure 2). The third most common complication, recorded in five cases (4.3%), was hemothorax, and four (80.0%) of those cases were considered mild (Figure 3). We defined complications as the presence of at least one of those three conditions (Table 3).

**Table 3:** Distribution of complications after CT-guided percutaneous biopsy of subcentimeter lung nodules.

Complication	Category	N	%^*^	*%^†^*
Alveolar hemorrhage	Mild	36	85.7	31.3
	Moderate	4	9.5	3.5
	Pronounced	2	4.8	1.7
	Total	42	100.0	36.5
Hemothorax	Mild	4	80.0	3.5
	Moderate	1	20.0	0.9
	Total	5	100.0	4.3
Pneumothorax	Mild	23	82.1	20.0
	Moderate	5	17.9	4.3
	Total	28	100.0	24.3

* Percentage of total observations.

† Percentage of the total number of subjects.

As can be seen in Table 4, no statistically significant differences in complications were observed in relation to sex (*p* = 0.58), nodule density (*p* = 0.79), lung segment (*p* = 0.19), age (*p* = 0.115), nodule size (*p* = 0.870), or distance traversed by the needle in lung tissue (*p* = 0.751). Our analysis also revealed no statistically significant relationship between the presence of a restrictive breathing pattern and the occurrence of pneumothorax (*p* = 0.124; chi-square test). The mean displacement in the parenchyma was 23.6 mm, ranging from 0 mm to 70 mm. However, we found no significant association between the length of the needle path in the parenchyma and the rate of pneumothorax (*p* = 0.512). We also found no significant association between nodule size and the occurrence of pneumothorax (*p* = 0.760). Likewise, the occurrence of complications in general was not found to show a significant association with the final results of the study (*p* = 0.984).

**Table 4:** Correlations of sex, density, and lung segment with the occurrence of complications.

Variable	Category	Complications		*P*
		No n (%)	Yes n (%)	
Sex	Female	31 (58.5)	32 (51.6)	0.582
	Male	22 (41.5)	30 (48.4)	
Density	Solid	34 (64.2)	43 (69.4)	0.797
	Semisolid	14 (26.4)	14 (22.6)	
	Ground glass	5 (9.4)	5 (8.1)	
Segment	Middle lobe and lingula	11(20.8)	11 (17.7)	0.195
	Lower lobes	29 (54.7)	26 (41.9)	
	Upper lobes	13 (24.5)	25 (40.3)	

## DISCUSSION

In all patients, the core needle technique and coaxial system, widely recommended as the first-line approach^**(12-14)**^, were used, thus reducing procedure time and complications. It has been shown to be superior to fine-needle aspiration cytology performed, because it provides information not only on the cytological characteristics of the lesion but also on the tissue architecture, which reduces the risk of inadequate sampling and allows the collection of multiple samples for histopathological, immunohistochemical, molecular, and genetic analyses, thereby enabling the targeting of specific therapies in the treatment of cancer^**(12,15)**^.

Biopsies of nodules ≤ 10 mm are challenging. Possible explanations for the wide-ranging results include the degree of difficulty of the procedure, the experience of the operator, the experience of the pathologist, and the persistence required in order to obtain a satisfactory tissue sample^**(12,14,16)**^.

Although CT-guided fine needle aspiration is widely used for the diagnosis of small lung lesions, studies have reported a reduction in diagnostic accuracy for smaller lesions. In addition, this approach is subject to variations in results, depending on the experience and availability of the pathology team during the procedure^**(13,16-20)**^.

The difficulty in obtaining a specific benign histological diagnosis depends on several factors, including the desire of the pathologist to exclude the possibility of malignancy, given that most lesions are biopsied for that purpose. An accurate diagnosis of an infectious process is only possible when the pathogenic agent is identified or when multinucleated cells with changes suggestive of viral inclusion are identified, making the request for cultures extremely important. In this scenario, diagnoses of benignity, as in cases of benign tumors such as hamartomas, which have classic histopathological characteristics, allow an experienced pathologist to make an accurate diagnosis^**(12)**^. Therefore, these techniques are especially critical in high-risk patients with significant cardiopulmonary diseases, in whom the impact of a repeat biopsy with a false-negative result or the occurrence of complications can be considerable^**(16)**^.

### Positioning

Optimal patient positioning is crucial because it seeks to ensure maximum comfort while providing an ideal access window and minimizing the risk of complications. In general, the patient should be positioned so that the entry into the skin is as short and vertical as possible, thus avoiding the occurrence of fissures and bullae. The supine position may be preferred in some cases, given that the movements of the posterior ribs are of smaller amplitude, making the intercostal access window more predictable^**(2)**^. In our study, we categorized the patient position in relation to the lesion as biopsy side down or biopsy side up. We observed no correlation between the patient position and the incidence of pneumothorax or other complications.

### Complications

The most common complications observed in this study were alveolar hemorrhage, with an incidence of 36.5%, followed by pneumothorax, with an incidence of 24.3%, and hemothorax, with an incidence of 4.3%. These results are similar to those of previous studies^**(12,14)**^.

The incidence of alveolar hemorrhage as a complication of CT-guided needle lung biopsy reported in the literature ranges from 3.4% to 43.0%^**(12,21)**^. In the present study, this complication occurred in 42 cases, of which 36 (85.7%) were classified as mild. Its incidence can be related to the difference in the length of the needle path used during biopsy material collection. One study showed that a path length of 10 mm results in a prevalence of alveolar hemorrhage of 14%^**(22)**^, whereas another study showed that a path length of 17-22 mm results in a 42% prevalence of the complication^**(23)**^. Alveolar hemorrhage can reduce diagnostic accuracy during biopsy procedures. However, in our study, this complication was mainly observed after the first hour post-biopsy, especially in cases of very small (< 10 mm) nodules. That delayed presentation can sometimes impair visualization of the lesion in subsequent follow-up assessments^**(12)**^. Among the cases that developed alveolar hemorrhage, the mean length of the needle path in the parenchyma was 21.2 mm, varying from a minimum of 0 mm to a maximum of 67 mm. We found no significant difference related to the length of the needle path in the parenchyma and the occurrence of alveolar hemorrhage (*p* = 0.930).

Another common complication after lung biopsy is pneumothorax, with reported incidences ranging from 12% to 65%^**(19,21,24-26)**^. The highest rates have been reported for lesions ≤ 10 mm^**(16,27)**^. In the present study, the incidence of pneumothorax was 24.3%, similar to that observed in previous studies^**(12)**^, and this relatively low incidence is probably related to the coaxial technique used, which minimizes the number of pleural punctures. In contrast, Lucidarme et al.^**(28)**^ reported a higher rate of pneumothorax, associated with lesions that required greater displacement of the lung parenchyma.

Greater distance between the lesion and the pleura is a well-documented risk factor for pneumothorax. Several studies have indicated that the rate of pneumothorax increases in parallel with an increase in the lesion-pleura distance^**(29,30)**^. It can be argued that a longer needle path during the procedure increases the likelihood of injury to the pleura and normal lung tissue, especially as the patient breathes. In the present study, the mean lesion-pleura distance was 23.6 mm, ranging from 0 mm to 70 mm; however, we did not find a significant association between that distance and the rate of pneumothorax. In contrast, Yeow et al.^**(21)**^ reported that the rate of pneumothorax was higher for intrapulmonary lesions located 10-20 mm from the surface of the pleura than for those at greater depths. That occurs due to superficial anchorage, which facilitates the displacement of the needle into the pleural cavity, resulting in air entry.

Gupta et al.^**(24)**^ found no statistically significant difference in the rate of pneumothorax among lesions with diameters ≤ 10 mm, 11-15 mm, and 16-20 mm. Similarly, we found no significant association between nodule size and the occurrence of pneumothorax.

Our results regarding the sensitivity and specificity of CT-guided core needle biopsies of pulmonary nodules are consistent with those of other studies^**(1,12-14,17,31,32)**^. The overall diagnostic accuracy of CT-guided lung biopsy in nodules ≤ 10 mm, using 18 G and 20 G needles, is remarkably high, with a 97.5% success rate in obtaining satisfactory material for analysis.

The present study presents the limitations typically associated with a retrospective study design. In some cases, we did not have access to all of the necessary data, either because the data were not recorded or because the patient was lost to follow-up.

Choosing a biopsy technique that minimizes patient exposure to radiation is essential to ensure safety. Therefore, an accurate biopsy of pulmonary nodules < 10 mm in diameter is extremely important, assuming that it is a reproducible method, well tolerated by patients and applicable in the diagnosis of benign and malignant lung lesions.
